# Checklist to operationalize measurement characteristics of patient-reported outcome measures

**DOI:** 10.1186/s13643-016-0307-4

**Published:** 2016-08-02

**Authors:** David O. Francis, Melissa L. McPheeters, Meaghan Noud, David F. Penson, Irene D. Feurer

**Affiliations:** 1 Department of Otolaryngology, Vanderbilt University Medical Center, Medical Center East, Suite 7302, 1215, 21st Avenue South, Nashville, TN 37212 USA; 2 Center for Surgical Quality and Outcomes Research, Institute for Medicine and Public Health, Vanderbilt University Medical Center, Nashville, 37232 TN USA; 3 Vanderbilt Evidence-based Practice Center, Institute for Medicine and Public Health, Vanderbilt University Medical Center, Nashville, 37232 TN USA; 4 Department of Health Policy, Vanderbilt University Medical Center, Nashville, 37232 TN USA; 5 Center of Population Science, Institute for Medicine and Public Health, Vanderbilt University Medical Center, Nashville, 37232 TN USA; 6 Departments of Urological Surgery and Medicine, Vanderbilt University Medical Center, Nashville, 37232 TN USA; 7 Geriatric Research Education and Clinical Center, Veterans Administration Tennessee Valley Healthcare System, Nashville, USA; 8 Departments of Surgery and Biostatistics, Vanderbilt University Medical Center, Nashville, 37232 TN USA

## Abstract

**Background:**

The purpose of this study was to advance a checklist of evaluative criteria designed to assess patient-reported outcome (PRO) measures’ developmental measurement properties and applicability, which can be used by systematic reviewers, researchers, and clinicians with a varied range of expertise in psychometric measure development methodology.

**Methods:**

A directed literature search was performed to identify original studies, textbooks, consensus guidelines, and published reports that propose criteria for assessing the quality of PRO measures. Recommendations from these sources were iteratively distilled into a checklist of key attributes. Preliminary items underwent evaluation through 24 cognitive interviews with clinicians and quantitative researchers. Six measurement theory methodological novices independently applied the final checklist to assess six PRO measures encompassing a variety of methods, applications, and clinical constructs. Agreement between novice and expert scores was assessed.

**Results:**

The distillation process yielded an 18-item checklist with six domains: (1) conceptual model, (2) content validity, (3) reliability, (4) construct validity, (5) scoring and interpretation, and (6) respondent burden and presentation. With minimal instruction, good agreement in checklist item ratings was achieved between quantitative researchers with expertise in measurement theory and less experienced clinicians (mean kappa 0.70; range 0.66–0.87).

**Conclusions:**

We present a simplified checklist that can help guide systematic reviewers, researchers, and clinicians with varied measurement theory expertise to evaluate the strengths and weakness of candidate PRO measures’ developmental properties and the appropriateness for specific applications.

**Electronic supplementary material:**

The online version of this article (doi:10.1186/s13643-016-0307-4) contains supplementary material, which is available to authorized users.

## Background

Improved health expectations have led to a shift away from viewing health in terms of survival toward defining as freedom from disease, followed by concentration on an individual’s ability to perform daily activities, and more recently to an emphasis on themes of well-being and quality of life [1–4]. Concomitant to the evolving conception of population health has been a transition from reliance on clinically focused end points without direct input from patients [5, 6] to increased emphasis on patient-centered outcome research and comparative effectiveness research [7]. As such, patients, families, and clinicians are increasingly faced with complex choices and ambiguous information when addressing health and healthcare needs.

It is important to differentiate between patient-centered *data* and patient-centered *outcomes*. Data are information deriving directly from patients, and outcomes are end points that matter to patients [6, 7]. A National Institutes of Health/Food and Drug Administration (FDA) working group identified three categories: *feeling*, *function*, and *survival* as primary patient-centered outcomes to be focused on and incorporated into all clinical trials proposing novel interventions, devices, or pharmaceuticals that aim for FDA approval [5]. A significant challenge in patient-centered outcome research and comparative effectiveness research is how best to identify and use patient-centered outcomes that measure effectiveness, facilitate decision-making, and inform health policy [8]. Patient-reported outcome (PRO) measures are now commonly used in this capacity and are defined as “any report on the status of a patient’s health condition that comes directly from the patient, without interpretation of the patient’s response by a clinician or anyone else” [8, 9].

Nomenclature in this field is nuanced and PROs, PRO measures, and health-related quality of life (HRQOL) are often used interchangeably [10, 11]. *Health-related quality of life* is “the value assigned to duration of life as modified by impairments, functional status, perceptions, and social opportunities that are influenced by disease, injury, treatment, or policy” [11–15]. In distinction, *PROs* provide reports *directly* from patients about health, quality of life, or functional status related to the healthcare or treatment they have received [6, 16], and *PRO measures* are designed to measure and report PRO constructs [6, 17]. We have chosen to use the term “PRO measure” heretofore to encompass the various types of health-related instruments including HRQOL, recognizing that others may prefer other terms [10, 16, 18]. Our rationale is that these types of instruments span a diverse gamut that include symptom indices [19, 20], general [21] and condition-specific HRQOL [22, 23], utilities [24, 25], well-being [26, 27], or social health [28] or can focus on latent constructs such as self-efficacy [29] and willingness to change [30, 31].

Patient-reported outcome measures address the need for patient-centered data and are now used in diverse clinical, research, and policy pursuits [32]. Greater emphasis on patient-centered care has resulted in instrument proliferation [33]. However, their developmental rigor and intended application vary widely [34], and this variation is likely to be reflected in systematic reviews. For instance, these instruments can be used as outcomes for group-level analyses in clinical trials and observational studies [35], but are also used to track within-person change over time [36], for group-level quality improvement initiatives to provide information for report cards [37], and as health surveys to monitor population health [38, 39]. In practice, a specific measure may be used in any or all these applications.

Patient-reported outcome measures have origins in various measurement theory-related disciplines including psychometrics [40], clinimetrics [41], and econometrics [4]. There is considerable overlap in approach between these disciplines, and collectively, they strengthen quantitative design methodologies. The common core principles of measure development are multifaceted and sometimes complex. Identifying the appropriate PRO measure for a particular purpose requires nuanced understanding of a candidate measure’s underlying conceptual model and its measurement properties [16]. Most clinicians, researchers, and patient advocates are not experts in the technical methods used to develop and validate these tools and may, understandably, presume similar performance among published PRO measures that address a particular construct. This is problematic since nearly all published tools purport some degree of these attributes, most often as forms of reliability or validity [34].

To address this issue, increased attention has been directed toward understanding what defines adequacy among PRO measures [5, 6, 10, 34, 42, 43]. This is directly relevant to systematic reviewers choosing to incorporate PRO measures as outcomes for their reviews. Current expert panel recommendations and proposed criteria on this topic have substantial homology, but differences do exist [6, 10, 34, 42–44]. Some advanced criteria are not easily understood, and others are rigorously prescriptive, tending to render most instruments inadequate in several respects. These concerns have contributed to disparate quality among systematic reviews of PRO measures and have the potential to mislead researchers into reliance on inappropriate or suboptimal instruments for a given purpose [10, 45–47]. For example, measurement bias in estimation of treatment effects can occur due to lack of conceptual equivalence between PRO measures [47].

An important and rigorous effort to aid researchers in the selection of appropriate PRO measures, the COnsensus-based Standards for the selection of health Measurement INstruments (COSMIN), was devised between 2006 and 2010 by an expert panel with diverse backgrounds (e.g., clinical medicine, biostatistics, psychology, epidemiology) [11, 16, 44, 46, 48]. Consensus was achieved as to measurement properties that should be assessed and on criteria for acceptable measurement [11, 16, 44]. Three overarching measurement domains were agreed upon: reliability, validity, and responsiveness. The product of this important work was a detailed algorithm for each identified domain. COSMIN remains the standard in the assessment of patient-reported outcome measures. However, its complexity (i.e., 119 items over 10 categories) may limit its utility for a systematic reviewer, researcher, or clinician who may not have expertise in measurement theory. Furthermore, its stated use is for evaluative instruments designed for applications to measure change over time. It may not apply for discriminative instruments, those used for predictive purposes, or healthcare-related measures used to measure satisfaction with care or adherence [16].

A simplified methodology that incorporates the critical features highlighted in COSMIN and other pertinent literature would be helpful to enable systematic reviewers, researchers, and clinicians to assess developmental characteristics and usefulness of a wide variety of PRO measures. In addition, its usefulness would be enhanced by the inclusion of practical aspects of PRO measures not consistently addressed in other criteria [6]. Thus, our study aimed to (1) advance a set of simplified criteria, in the form of a checklist, that can aid in systematically assessing the measurement properties and usefulness of PRO measures for particular circumstances and 2) demonstrate the checklist’s user-friendliness by determining the inter-rater reliability of its scoring between clinicians/researchers with and without expertise in empirical instrument developmental methods. The resultant checklist is intended as a guide for systematic reviewers, researchers, and clinicians with diverse measurement theory expertise to aid in identifying the strengths, weaknesses, and applicability of candidate PRO measures.

## Methods

A review of the literature was performed to identify recommendations for evaluating PRO measures. The directed search enabled the compilation of PRO measures’ developmental recommendations from a wide variety of sources including the FDA [5, 6], the Scientific Advisory Committee of the Medical Outcomes Trust [43, 49], COSMIN [11, 16, 44, 46], Agency for Healthcare Research and Quality [10], American Psychological Association [50, 51], measurement theory textbooks [40, 52–54], and individual studies via a PubMed search for evaluative criteria germane to PRO measures, health-related quality of life, and related terminology. This study did not involve data collection from or about human subjects and was therefore exempt from IRB review.

Two investigators (DOF, IDF) analyzed and synthesized these recommendations and iteratively distilled them into initial criteria. Attributes considered fundamental were (1) conceptual model, (2) content validity, (3) reliability, (4) construct validity, (5) scoring and interpretation, and (6) respondent burden and presentation. Founded in psychometrics (e.g., classical test and item response theories) [40, 43, 55, 56] and clinimetrics [57], the core qualities outlined below encompass the theoretical underpinnings of a PRO measure and the developmental characteristics necessary to ensure its overall usefulness.

1. *Conceptual model* provides a rationale for and description of the concepts and the populations that a measure is intended to assess [8, 43, 58, 59]. The *concept* is the specific measurement goal and should be explicitly stated in the development process. Conceptual models are developed by outlining hypothesized and potential concepts and relationships and by determining the target population and model’s application [6, 58, 60]. In assessing its adequacy, a candidate measure’s original development should be examined to determine if it is likely to capture the intended effect [10]. Whether multiple domains or subscales are expected should be clearly inherent to or directly pre-specified within the conceptual framework [8, 43, 61]. Ninety percent of International Society for Quality of Life Research survey respondents endorsed that PRO measures should have documentation defining the construct and describing the measure’s application in the intended population [5, 8, 43].
2. *Content validity* refers to evidence that a PRO measure’s domain(s) is appropriate for its intended use in both relevance and comprehensiveness [10, 43, 46, 61, 62]. No formal statistical test exists to evaluate content validity. Instead, assessment is done through applying qualitative criteria. Specifically, items (i.e., questions) and conceptual domains (e.g., subscales) should be relevant to target patients’ concerns. Thus, developers should obtain input from the target population to optimize item relevance and clarity, ideally, through qualitative focus groups and cognitive interviews [5, 61]. In brief, cognitive interviews are a qualitative research tool used to determine whether respondents understand included concepts and items in the way that PRO measure developers intend. These interactive “field-test” interviews allow developers to better understand how respondents interpret candidate questions [6]. Similarly, content experts should participate in PRO measure development with emphasis on evaluating the relevance of items for the construct and for the respondent population [43, 46, 61, 62], and there should be a thorough description of how items were elicited, selected, and developed [5].
3. *Reliability* is the degree to which scores are free from random (measurement) error [11, 43]. Several forms exist. *Internal consistency reliability*, the degree to which segments of a test (e.g., split halves, individual items) are associated with each other [56], reflects precision at a single time point [43]. It is based on correlation of scores between different items within the PRO measure, thus assessing whether items proposed to measure the same general construct or domain are statistically related. *Test-retest reliability* refers to the reproducibility or stability of scores over two administrations, typically in close temporal proximity, among respondents who are assumed not to have changed on the relevant domains [43, 56]. Traditionally cited minimum levels for reliability coefficients are 0.70 for group-level comparisons and 0.90 to 0.95 for individual comparisons [8, 43]. Coefficients indicate the ratio of true score variance to observed score variance. These thresholds are important to establish the reliability of an instrument. However, some argue that establishing absolute thresholds for interpreting coefficients may be overly prescriptive [8, 53]. Therefore, reliability estimates lower than the convention cited above should be justified in the context of the proposed PRO measure’s intended application, its sample size, and the reliability statistic used [63].
4. *Construct validity* refers to whether a test measures theoretic intended constructs or traits [40, 43, 56], and it directly affects the appropriateness of measurement-based inferences. Evidence of construct validity can derive from empirical demonstrations of dimensionality [5, 55]. A variety of latent variable modeling techniques such as factor analysis are available to evaluate and provide evidence of dimensionality, and these methods should be used and reported when subscales or domains are proposed or expected. Factor analysis (and related latent variable methods) is, in general, a data reduction method intended to mathematically represent a large number of differentially related questions (i.e., items) by a smaller number of latent dimensions or “factors.” A factor is a mathematical representation of a collection of variables that are statistically related to one another, which differs conceptually from other factors [53, 55]. Generally speaking, factor analysis methods such as common factor analysis, principal components analysis, and bi-factor analysis are important in both classical and item response theory-based instrument development processes [55].
  *Responsiveness to change*, which is also known as longitudinal construct validity [64], can be considered an aspect of validity [65] or as a separate dimension [11]. It is the extent to which a PRO measure detects meaningful change over time when it is known to have occurred [8, 43, 66]. Most, but not all, instruments have a stated goal of measuring change over time. Thus, this property is not applicable to PRO measures intended specifically for cross-sectional study designs. If a measure is not intended to measure change (e.g., screening test), this point should be specified in the conceptual model. Responsiveness requires demonstrable test-retest reliability *and* the ability to detect an expected change (e.g., after intervention) in the intended population [43, 66]. Absence of either element limits the confidence that measured differences in scores represent an actual change rather than measurement error.
  Responsiveness to change can be measured using two approaches: distribution- or anchor-based methods. Distribution-based methods are based on either within-group change over time or between-group comparisons. Such approaches are characterized by an effect size, standard response mean, or as other measures that account for actual change related to random error (e.g., standard error of measurement) [10]. Anchor-based methods quantify differences by examining the relationship between the PRO measure score and an independent measure (anchor) that could be patient-based, physician-based, or an alternate external assessment of construct severity [67–69]. Both methodologies necessarily incorporate both expected change and test-retest reliability in their calculation. Candidate PRO measures’ responsiveness characteristics are particularly relevant for systematic reviewers aiming to compare effectiveness of interventions.
  Another form of construct validity is the degree to which PRO measure scores correlate with other questionnaires that evaluate the same construct or with related clinical indicators (e.g., pulmonary function tests) [43, 56]. This is sometimes referred to as “convergent validity.” A priori hypotheses about expected associations between a PRO measure and similar or dissimilar measures should be documented [8, 43]. A closely related concept, called “known groups” or *divergent validity*, requires the PRO measure to differentiate between groups that past empirical evidence has shown to be different. These types of validity have also been classified under the auspices of hypotheses testing [46].
  It is rarely possible to establish a PRO measure’s criterion validity because, in the majority of cases, no “gold standard” exists to measure the targeted construct [8]. It is, however, a pertinent parameter in questionnaires designed to be predictive of a certain state (*predictive validity*). For example, self-rated health has been shown to predict mortality [70]; thus, predictive validity can be considered a form of *criterion-related validity*. A clear distinction needs to be made between predictive and longitudinal validity (responsiveness). The former refers to the ability of a “baseline” score (e.g., test result) to predict some future event [53] and is reflected by that association. It does not imply a measure’s ability to distinguish change between initial and follow-up assessments.
5. *Scoring and interpretation. Interpretability* is the degree to which the meaning of scores is easily understood [5, 8, 43, 71]. This requires that a *scoring system* be clearly described and that some form of *scaling* exists to indicate what different scores mean. A scoring system defines how to compute scores, whether as a total score or subscales, on the basis of empirical evidence (e.g., a principal component structure supporting a particular number of subscales). Scaling properties depend on the context of the measurement instrument. Total score and item-level scaling are often used and several methodologies exist, including those from classical test theory (e.g., standard error of true scores) [55, 56] and item response theory (e.g., Rasch modeling) [55, 56]. Empirically based scaling allows end users to readily interpret scores, as does considering the availability of relevant population-level or condition-specific normative data or “norms,” which permit referencing scores to appropriate standards.
  It is important to understand what represents a minimally important difference and to have the ability to differentiate degrees of difference (e.g., severity) for the construct [72]. Minimally important difference (MID) is defined as “the smallest difference in score in the outcome of interest that informed patients or proxies perceive as important, either beneficial or harmful, and that would lead the patient or clinician to consider a change in management” [73]. In brief, MID can be established using distribution- or anchor-based approaches. The anchor should be independently interpretable, and there must be reasonable correlation between the PRO measure score and anchor [72, 74]. The distribution-based method uses the magnitude of change compared to the variability in scores (e.g., effect size). A salient argument is also made that the term “patient-important” is more appropriate than “clinically important” to emphasize the patient-centrism of these outcomes and the goals of directed interventions [18, 75]. The meaningfulness of differences should ideally be based on what patients consider a minimally important, small, moderate, and large difference [76]. Incorporating patients’ perspective on what constitutes a difference strengthens the clinical usefulness of the PRO measure. Without this information, it can be difficult to contextualize longitudinal or cross-sectional outcomes and understand if the magnitude of change is important.
  Finally, an often overlooked aspect of scoring and interpretability is an explicit plan for *managing and/or interpreting missing responses* [77], which are common in the practical use of PRO measures [78]. Missing item data introduces error in individual score computation. Data that are missing in a systematic manner may introduce bias into group- and population-level analyses. Several methods exist to manage missing responses and data, and instructions regarding how to manage missing responses are important. Without them, the user is often left to score only those surveys for which responses are complete.
6. *Respondent burden and presentation. Burden* refers to the time, effort, and other demands placed on respondents or those administering the instrument [43, 71]. Acceptable burden in the context of the number of items in and the time necessary to complete a PRO measure is somewhat subjective and depends on the measure’s intended use. Lengthy measures might be considered reasonable in a research setting but overly burdensome if administered during a busy clinic. These issues should be explicitly considered, as overly burdensome PRO measures can limit their applicability and practical adoption into studies [79]. The length of a PRO measure should be contextually appropriate [71].
  Another consideration of burden and presentation is the *literacy level* required to understand and complete the measure [80]. Most experts recommend that items be at the sixth grade reading level or lower; however, this criterion should be contextualized to the intended target population [8] and it should be justified. Finally, a PRO measure’s items and their presentation should be available to be viewed or accessed by persons considering incorporating its use into practice [71]. Without this level of transparency, it is difficult to fully evaluate a prospective instrument’s appropriateness for a particular application.

### Cognitive interviews

**Fig. 1:**
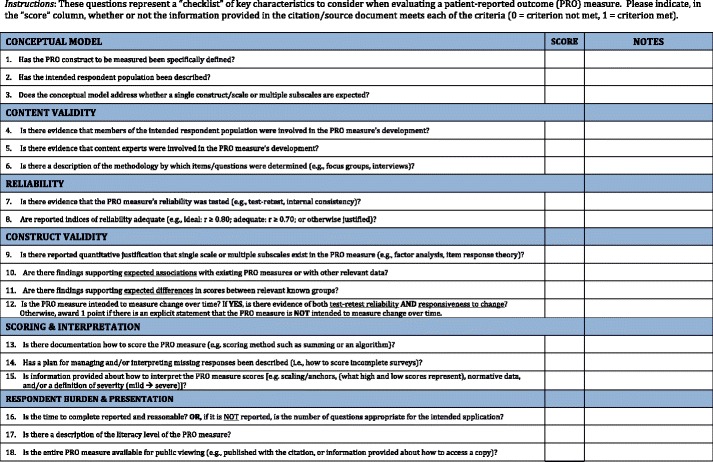
Checklist to operationalize developmental characteristics and applicability of patient-reported outcome measures

Our goal of distilling key criteria into a checklist was to provide guidance on how to systematically evaluate candidate PRO measures’ developmental characteristics and usefulness for a particular purpose. The intended audience for the proposed criteria is systematic reviewers, researchers, and clinicians with varied expertise in PRO measure development and application. Thus, the initial criteria checklist was reviewed by a group of 12 clinicians (medical students [*n* = 3], physicians [*n* = 9]) and 12 investigators with expertise in survey-based quantitative methods (MPH [*n* = 6], PhD/DrPH [*n* = 6]). Each participant was asked to review and comment on the clarity, accuracy, completeness, and user-friendliness of the criteria. Study personnel asked respondents directed follow-up questions to foster discussion and further clarification of concerns. Comments were used to improve clarity, readability, accuracy, and completeness and to establish the revised final criteria checklist (Fig. 1).

### Inter-rater reliability of the checklist

Two investigators (DOF, IDF) used the checklist to assess six pre-specified PRO measures encompassing a variety of methods and applications related to voice and swallowing disorders [81–86]. Two measures were designed to measure handicap (VHI, VHI-10) [81, 82], and one each was designed to measure health-related quality of life (V-RQOL) [85], coping (VDCQ) [84], and activity and participation (VAPP) [87] associated with voice disorders. Another measure developed using item response theory techniques focused on health-related quality of life among patients with achalasia [86]. Discordances were resolved with a modified Delphi technique, and agreed upon criterion-level decisions and tallies provided reference values for each measure. A group of six clinicians without expertise in measurement theory graded the six PRO measures, and their agreement with reference “scores” was summarized as the kappa statistic. An a priori threshold for kappa was set at greater than 0.50 for each PRO measures to demonstrate at least moderate agreement. A stepwise process was used. Participating clinicians were first provided with the checklist (Fig. 1) and brief written descriptions of concepts being evaluated (Additional file 1: Figure S1). Each independently scored the PRO measures, and if kappa scores were inadequate, participants were provided 15 min of individualized education on the concepts followed by rescoring as necessary. This process provided more in-depth information and detailed feedback regarding parameters included in the criteria.

## Results

### Cognitive interviews

The cognitive interviews highlighted that several respondents were concerned that the checklist mentioned technical detail or sophisticated concepts that the average user would not be familiar with (e.g., factor analysis, item response theory). Responding to these concerns, an addendum was created and appended (Additional file 1: Figure S1).

Respondents expressed concern that some criteria did not have strict benchmarks for decision-making. An example is “has the PRO construct been specifically defined?” Supporting documentation was clarified to note that these criteria are necessarily general (and somewhat vague) due to their inherent subjectivity and absence of specific standards. One respondent questioned whether the target population’s demographic or clinical characteristics should be defined, and several recommended simplifying grammar and sentence structure. They unanimously questioned the propriety of summing the criteria into a total score and felt that the individual criteria presented did not warrant uniform weights.

Some respondents also recommended removing strict thresholds for interpreting reliability. Despite this recommendation, we opted to include them because they represent important, accepted conventions, especially since less experienced users need some guidance regarding interpretation. Some respondents felt it would be helpful to parenthetically list types of reliability that should be tested (e.g., test-retest reliability, internal consistency), and some questioned whether testing dimensionality through factor analysis or other quantitative approaches should be classified as a component of reliability rather than validity. While the characteristics of scales and the items comprising them can be assessed for their internal consistency reliability, we opted to present this concept in the construct validity section, with the rationale that empirically identified dimensions should reflect the conceptual domains represented by the PRO measure.

Another characteristic that proved difficult for some respondents related to responsiveness. This question required that the PRO measure demonstrate both test-retest reliability *and* evidence of responsiveness to change. The rationale for the prerequisite of test-retest reliability was that if a PRO measure has not shown stability then evidence of responsiveness cannot be proven. Several reviewers suggested splitting this question so that it only takes into account responsiveness to change. Others recommended using the term “changes over time” rather than responsiveness or longitudinal validity.

Several persons recognized the subjectivity of asking reviewers to assess whether the PRO measure length was “reasonable.” Initially, an example length of 10 items was included if no mention of burden was mentioned. However, most respondents felt that was too prescriptive and that longer measures were not overly burdensome in specific circumstances. There was also question whether the ability to access the entire PRO measure really mattered. All of these issues were carefully considered, and many suggestions were incorporated in the final criteria.

### Proposed checklist

Shown in Fig. 1 is the proposed criteria checklist for assessing the development characteristics and utility of PRO measures. Eighteen characteristics are to be scored dichotomously (present/absent) in six general domains: conceptual model (three items), content validity (three items), reliability (two items), construct validity (four items), scoring and interpretability (three items), and respondent burden and presentation (three items). On the basis of feedback from cognitive interviews and in consideration of the stage of the instrument’s development, individual characteristics and domains were not weighted. The final criteria are referred to as a checklist and are intended as a guide when selecting or evaluating PRO measure.

### Agreement between novice users and reference scores

All six participating clinicians independently scored the same six individual PRO measures (*n* = 36). Overall, the mean clinician kappa for the first iteration (written instruction only) was 0.54 (range 0.35–0.63) with 21/36 reviews meeting the a priori criterion of kappa greater than 0.50. One clinician met the criterion on all six PRO measures on the first attempt (Table 1). Two participants met the threshold for 5/6, and one each met the threshold for 3/6, 2/6, and 1/6 of tested PRO measures.

**Table 1:** Interventions and novice reviewer agreement with reference scores

Reviewer	Written	Kappa (range)	1st teaching	Kappa (range)	2nd teaching	Kappa (range)
1	X	0.63 (0.50–0.82)				
2	X	0.59 (0.25–0.77)	X	0.66 (0.56–0.77)		
3	X	0.34 (0.02–0.61)	X	0.66 (0.56–0.82)		
4	X	0.51 (0.27–1.00)	X	0.72 (0.54–1.00)		
5	X	0.64 (0.49–1.00)	X	0.66 (0.51–1.00)		
6	X	0.54 (0.09–0.85)	X	0.65 (0.33–1.00)	X	0.87 (0.68–1.00)

X indicates that this type of instruction was provided to the reviewer. First provided was written instructions followed by in-person teaching (teaching was ≤15 min). Kappa scores: mean (range)

The five remaining participants each received brief education on concepts and rescored the measures for which agreement was below the criterion (*n* = 15). In the second iteration, four of five participants achieved adequate agreement on all measures (Table 1). One required a second educational session, followed by rescoring, and thereafter achieved adequate agreement on all PRO measures. The final mean kappa statistic for the clinicians was 0.70 (range 0.66–0.87; Table 1).

## Discussion

We distilled existing consensus criteria into a checklist that can be readily employed in systematic reviews that aim to assess PRO measures’ developmental properties. This checklist provides end users a means to evaluate the appropriateness of PRO measures prior to applying them for research or clinical purposes. The checklist’s strength is the demonstration that, with minimal instruction, systematic reviewers, researchers, and clinicians with limited PRO measure methodological expertise can apply it with ratings that correlate highly with experts in instrument development methodology.

There are long-standing discussions about what constitutes quality among survey and test instruments that even occur in the fields of psychology and education, where measurement theory was initially developed and promulgated. An initial consensus statement in 1954 identified the core qualities of survey development as dissemination, interpretation, validity, reliability, administration and scoring, scaling, and norms [50]. Social scientists, statisticians, and health outcome researchers have refined and advanced these developmental methodologies; however, the same principles first described still pervade consensus statements and expert opinion in the fields of education, social science, and healthcare.

Incorporation of PRO measures’ developmental methodology in healthcare has evolved rapidly with the emergence of comparative effectiveness research and patient-centered outcome research. Feinstein aptly described the foundation of this important work stating that “assessment of health status is important because improvements in symptoms, other clinical problems, and functional capacity are usually the main goals of patients in seeking care” [88]. Patient-reported outcome measures are increasingly used to better understand the perspectives of and to measure concepts that matter to the patient [5]. Methodological experts in PRO measures and survey design have disseminated several consensus statements to guide appropriate development and implementation of these measures [5, 8, 10, 43, 89]. Use of poorly developed PRO measures or those designed for a purpose that differs from their use can have significant implications and lead to distorted, inaccurate, or equivocal findings [5, 47]. Measures should be chosen based on relevance and their track record in the context of the proposed study [10]. Therefore, it is incumbent upon researchers and other end users to carefully consider a measure’s properties and weigh its strengths and potential weaknesses before implementing it in practice, clinical trials, quality improvement initiatives, or population-level studies.

Simplified access to evaluation criteria should encourage easier and more careful vetting of candidate PRO measures by potential end users. It can be applied to evaluate a specific instrument’s characteristics or in the performance of systematic reviews of PRO measures’ developmental properties. The complexity and prescriptiveness of prior consensus guidelines on PRO measure development may limit their practical application by systematic reviewers, researchers, and clinician end users who are not expert in survey design and measurement theory. To overcome this issue, we have advanced a simple checklist for evaluating the adequacy of any survey or PRO measure. It cannot be over emphasized that its contents are not intended to replace prior consensus statements on this topic. Instead, it aims to distill and harmonize homologous concepts that have been widely recognized in published expert consensus statements.

### Considerations and limitations

Our proposed checklist is not exhaustive. Psychometric and clinimetric PRO measures’ development principles are often complex, conceptually overlapping, and evolving [90]. It is not possible to accommodate and incorporate all parameters and circumstantial caveats within simple criteria. One example is administrative burden (e.g., personnel time needed to help patients complete questions), which can affect the ease of application of a particular PRO measure and was not explicitly addressed in the present checklist. Further, it is important to recognize that the fundamental principles of survey development exist on a spectrum, are often interchangeable, and are not necessarily discrete concepts. An example is responsiveness, which has been categorized as an aspect of validity [64, 65] but also as its own domain [11]. Additionally, because each checklist characteristic was derived from broadly accepted core concepts in survey methodology and measurement theory that by their nature are not necessarily expected to correlate with each other, the utility of latent variable methods such as factor analysis are not applicable at this stage.

The relative importance of a specific measurement property may vary substantially with the purpose and context of a PRO measure’s use. As such, we do not recommend a total score for this tool since this implies each item should be weighted equally. Our analysis of inter-rater reliability of ratings between novice and more experienced practitioners of measurement theory was not intended to provide rigorous evidence of the checklist’s completeness. Instead, this preliminary analysis was performed to show that this simple checklist was easy to apply and reliable even among those with little expertise the field. The proposed system is designed to serve as a guide to understand the strengths and weaknesses and applicability of any particular survey or PRO measure.

## Conclusions

Systematic reviewers, researchers, and clinicians who are considering using a particular PRO measure as an outcome in the performance of or evaluating clinical trial results need to be able to assess whether the instrument used was appropriate for the intended use. The checklist provides simplified criteria that can be used to assess developmental properties and usefulness of a variety of PRO measures by end users with a wide range of expertise in measurement theory, psychometrics, or survey development. Our intent was not to replace the currently available comprehensive evaluative consensus guidelines. Instead, we propose that these criteria serve as a distilled and simplified version of characteristics that constitute an adequately developed PRO measure. Psychometricians, statisticians, measurement theory experts, econometricians, and clinicians have iteratively developed and discussed these properties over decades, in a literature that encompasses an array of disciplines. Refinements and evolution of these techniques continue. However, the general fundamentals remain the bedrock on which these innovations build. Our criteria attempt to summarize these foundational concepts into a user-friendly checklist that will help end users with a variety of backgrounds to identify the strengths and weaknesses of available PRO measures for their particular application.

## Additional file

Additional file 1: Written description of patient-reported outcome measure concepts included in the checklist. (DOCX 16.0 MB)

## Abbreviations

PRO: Patient-reported outcome
FDA: Food and Drug Administration
HRQOL: Health-related quality of life
COSMIN: COnsensus-based Standards for the selection of health Measurement INstruments
MID: Minimally important difference
MPH: Masters of Public Health
PhD: Doctor of Philosophy
DrPH: Doctor of Public Health
