# Supplementary material for: Checklist to operationalize measurement characteristics of patient-reported outcome measures
Source: Syst Rev. 2016 Aug 2;5(1):129. doi: 10.1186/s13643-016-0307-4 (PMC4971647; doi:10.1186/s13643-016-0307-4)
Supplement: Additional file 1: — Written description of patient-reported outcome measure concepts included in the checklist. (DOCX 16.0 MB) [file 13643_2016_307_MOESM1_ESM.docx]

**Figure 2**. Written description of patient reported outcome measure concepts included in checklist

**Conceptual Model**

1. **Has the PRO construct to be measured been specifically defined?**

The goal of PRO measures is to measure a construct (i.e., concept). This must be defined clearly before developing the measure. Examples of constructs include intelligence, disability, and anxiety.

1. **Has the intended respondent population been described?**

The population that is targeted for measurement should be clearly described. An example is a PRO measure trying to evaluate disability in patients with depression.

1. **Does the conceptual model address whether a single scale or multiple subscales are expected?**

Some measures intend to measure a single concept (i.e., pain) whereas others intend to measure multiple concepts. For example, a PRO measure of intelligence may **expect** *a priori* to have separate subscales measuring 1) emotional, 2) mathematical, and 3) spatial intelligence.

**Content Validity**

1. **Is there evidence that members of the respondent population were involved in the development of the PRO measure?**

Participants targeted by the PRO instrument should be intimately involved in its developmental process. This should be explicitly stated in the manuscript. For example, patients with depression should be included and their perspectives incorporated when developing a PRO measure aiming to quantify depression.

1. **Is there evidence that content experts were involved in development of the PRO measure**?

Input from experts that care for or have specific expertise in the construct area should be included and their perspectives incorporated into the development of the patient reported outcome measure. An example would be psychiatrists who treat patients with depression being involved in the development of PRO measure of depression.

1. **Is there a description of the methodology by which items/questions were derived?**

It should be explicitly stated how the items or questions were derived. Specifically, this should address how the respondent population and content experts were accessed and ultimately how questions/items in the PRO measure were created from this process. Examples include focus groups and interviews of respondents or content experts.

**Reliability**

1. **Is there evidence that the reliability of the PRO measure was tested (e.g., test-retest, internal consistency)?**

It is important that there has been some mention that reliability was tested during the development process. Examples of reliability include test-retest and internal consistency reliability.

1. **Are reported indices of reliability adequate (e.g., ideal: r>=0.80; adequate r>=0.70; or lower if justified)?**

The authors should provide some quantification of degree of reliability and that documented should meet those thresholds listed or should be otherwise justified.

**Construct Validity**

1. **Is there reported mathematical justification that a single scale or multiple subscales exist in the PRO measure (e.g., factor analysis, item response theory)?**

It is important that if authors claim to have multiple subscales in the PRO measure that they empirically demonstrate their existence. For example, if a PRO measure says they have 3 subscales: 1) emotional, 2) physical, 3) functional; then some mathematical principle should be applied to show commonness of items within each subscale and difference from other subscales. Examples of approaches to test for unique scales include factor analysis and item response theory (IRT) techniques.

1. **Is the PRO measure intended to measure change over time? If yes, is there evidence of both test-retest reliability and responsiveness to change? Otherwise, award 1 point if there is an explicit statement that PRO measure is NOT intended to measure change over time.**

It is important that PRO measures whose goal is to measure change over time show evidence of stability of score when no change is expected. Instability of scores will make identifying “real” from random or “chance” differences difficult. This stability is assessed using test-retest reliability technique. Secondly, evidence is also needed that the PRO measure changed meaningfully in an expected direction after an intervention (responsiveness to change). This can be established using anchor- or distribution-based approaches.

1. **Are there findings supporting expected correlations with existing PRO measures or other clinical data?**

It is important that the PRO measure scores correlate in an expected way with either an existing PRO measure(s) or clinical data that quantify the same concept (e.g., convergent validity).

1. **Are there findings supporting expected differences in scores between known groups?**

The PRO measure scores should be able to differentiate respondents who are expected to differ. For example, a measure of depression should be able to identify those with depression compared to those that do not (controls).

**Scoring & Interpretation**

1. **Is there documentation how to score the PRO measure?**

There should be a clearly explained scoring approach or algorithm.

1. **Has a plan for managing and/or interpreting missing responses been described?**

The authors should explain a method to deal with PRO measures that are not fully completed.

1. **Is there information provided on how to interpret the PRO measure scores?**

It is important that information is available to interpret PRO measure scores. For example, are there thresholds or explanations for what would indicate a mild, moderate, severe degree of the construct being measured?

**Respondent Burden & Presentation**

1. **Is time to complete reported and reasonable? If not, are number of questions appropriate for the intended application?**

Authors should indicate and justify the time necessary to complete the PRO measure. Those that do not include this information put the onus on the reviewer to determine whether the burden of time needed to complete the PRO measure is appropriate considering its intended application. For example, those intended for use in a busy clinical setting may need to be shorter than those used purely for research purposes.

1. **Is there a description of the literacy level of the PRO measure?**

It is important that the PRO measure is written at a level that intended respondents can understand. This should, at a minimum, be described in the manuscript. Most believe that a 6^th^ grade reading level is appropriate, but different education-reading levels may be more appropriate.

1. **Is the entire PRO measure available for public viewing?**

It is important to be able to access the PRO measure items/questions in order to evaluate the appropriateness of the questions and to assess the applicability for a particular purpose.
